# Development of a Website Providing Evidence-Based Information About Nutrition and Cancer: Fighting Fiction and Supporting Facts Online

**DOI:** 10.2196/resprot.4757

**Published:** 2015-09-08

**Authors:** Merel Rebecca van Veen, Sandra Beijer, Anika Maria Alberdina Adriaans, Jeanne Vogel-Boezeman, Ellen Kampman

**Affiliations:** ^1^ Netherlands Comprehensive Cancer Organisation (IKNL) Eindhoven Netherlands; ^2^ Wageningen University Division of Human Nutrition Wageningen Netherlands; ^3^ Dutch Dietetic Oncology Group Tilburg Netherlands; ^4^ VU University Amsterdam Netherlands; ^5^ Radboud University Medical Centre Nijmegen Netherlands

**Keywords:** cancer, information needs, Internet, nutrition, website development

## Abstract

**Background:**

Although widely available, the general public, cancer patients, and cancer survivors have difficulties accessing evidence-based information on nutrition and cancer. It is challenging to distinguish myths from facts, and sometimes conflicting information can be found in different places. The public and patients would benefit from evidence-based, correct, and clear information from an easily recognizable source.

**Objective:**

The aim of this project is to make scientific information available for the general public, cancer patients, and cancer survivors through a website. The aim of this paper is to describe and evaluate the development of the website as well as related statistics 1st year after its launch.

**Methods:**

To develop the initial content for the website, the website was filled with answers to frequently asked questions provided by cancer organizations and the Dutch Dietetic Oncology Group, and by responding to various fiction and facts published in the media. The website was organized into 3 parts, namely, nutrition before (prevention), during, and after cancer therapy; an opportunity for visitors to submit specific questions regarding nutrition and cancer was included. The website was pretested by patients, health care professionals, and communication experts. After launching the website, visitors’ questions were answered by nutritional scientists and dieticians with evidence- or eminence-based information on nutrition and cancer. Once the website was live, question categories and website statistics were recorded.

**Results:**

Before launch, the key areas for improvement, such as navigation, categorization, and missing information, were identified and adjusted. In the 1st year after the launch, 90,111 individuals visited the website, and 404 questions were submitted on nutrition and cancer. Most of the questions were on cancer prevention and nutrition during the treatment of cancer.

**Conclusions:**

The website provides access to evidence- and eminence-based information on nutrition and cancer. As can be concluded from the number of visitors and the number of questions submitted to the website, the website fills a gap.

## Introduction

### Background

With more than 10,000 epidemiological studies, and several animal and in vitro experiments, the major diet- and nutrition-related factors in the etiology of cancer are now well-known to researchers. Numerous reports and guidelines have been written on cancers, which are widely available both online and in print [1-6]. However, one of the most consistent findings in health services research is that scientific knowledge often does not reach the general public.

Although several studies have shown that adhering to the guidelines concerning a healthy weight, a healthy diet, and sufficient physical activity does reduce cancer risk [7,8], approximately one half to two thirds of people think these factors do not contribute to their cancer risk [9,10], despite the fact that these guidelines are actively addressed by both national and international organizations in their campaigns for the general public [2,11].

Not only are the guidelines to reduce the risk of cancer not well-known, but also cancer patients are not well informed about the guidelines regarding nutrition during and after cancer treatment. Because these guidelines are not sufficiently implemented in daily practice [12], this can result in inefficient, inappropriate, or even harmful care [13]. Furthermore, information about nutrition and cancer is often provided on request and is not routinely discussed during consultation [14]. The literature shows that there is a high demand for nutritional information among cancer patients [15], and that 30-66% of cancer patients have unmet nutritional information needs [15-20]. As a result, cancer patients and survivors try to fulfill these needs by finding information. In a survey conducted among 217 young adult cancer patients, almost 90% reported the need for nutritional information, and 95% of the respondents have used websites to search for information [15]. In a study involving around 2000 breast, prostate, and colon cancer patients, approximately 25% of patients reported searching for information online [21]. The monitoring of Internet use in The Netherlands in 2014 showed that 50% of health care users searched the Internet for information on nutrition and exercise [22].

The actual information on nutrition and cancer available online is overwhelming, with numerous websites from individuals, foundations, and industry and health care organizations providing information [2,11,23-28]. However, for the visitors of these websites, it may be hard to distinguish evidence-based websites from those that are not and to separate myths from facts. This may lead to misconceptions resulting in unnecessary or worse changes in dietary habits with a negative effect on nutritional status and response to treatment [29]. Furthermore, conflicting information leads to confusion and uncertainty, which may negatively influence the quality of life of cancer patients. The provision of appropriate information can result in an improved health competence, a better sense of control over cancer, better symptom management, lower levels of distress, and higher levels of health-related quality of life [30].

A recent study shows that active information seeking about cancer from nonclinical sources may lead to improved dietary habits among cancer patients [31]. Furthermore, an evidence-based website may lead to a better sense of control over cancer. A recently launched website for lung cancer patients was used by patients to better understand the information given by their own specialist. An evaluation of the site showed that access to this information helped them to better cope with their disease [32].

### Objective

Thus, cancer patients, both during and after treatment, as well as the general public, would benefit from correct, clear, and evidence-based information from an easily recognizable and evidence-based source. Therefore, the aim of this project is to make scientific information available to all people who have to deal with cancer. Because of the aforementioned positive effects, a website is used to provide people with nutritional advice to prevent cancer, nutritional advice during cancer treatment, and advice about what to do once their treatment is finished. The aim of this paper is to describe and evaluate the development of the website and to describe the experiences of the 1st year after the launch.

## Methods

### Prelaunch

The first content of the website Voeding en Kanker Info (Nutrition and Cancer Info) [33] was composed by collecting (1) items from oncology dieticians in daily practice (members of the Dutch Dietetic Oncology Group); (2) questions received by the helplines of various cancer organizations (eg, the Dutch Cancer Society and the World Cancer Research Fund); and (3) various fiction and facts published in the media.

### Answering Questions

Questions were answered by registered dieticians specializing in oncology and nutritional scientists and were reviewed by members of the Dutch Dietetic Oncology Group. Evidence from meta-analyses of observational studies, reviews, and randomized controlled trials (RCTs), in combination with evidence from animal and in vitro studies, was used to formulate those answers. All questions and answers were used as content for the website.

### Design

The website was designed with a home page where the questions were displayed. The editorial board, including the authors of the website, was fully shown on the home page. The 5 most prevalent tumor types (lung, breast, prostate, colorectal, and skin tumors) were described on separate pages; questions were categorized by tumor type and by the following categories: prevention, during treatment, or after treatment/tertiary prevention.

### Pretest

Before the official launch, a pretest was performed, and the website was evaluated by cancer survivors, health care professionals, and communication experts. Survivors were recruited from the Online Cancer Patient Panel of the Netherlands Comprehensive Cancer Organization and from the oncology day-care center of a peripheral hospital where the questionnaire was filled out while the patients received chemotherapy. The professionals were communication specialists, registered dieticians, nutritional scientists, the staff of the World Cancer Research Fund and the Dutch Cancer Society, and patient advocates. Those who tested the website filled out a questionnaire on site design and layout, content, readability, and comprehensiveness of the texts. The questionnaire contained both open-ended and closed questions. The questions asked in the questionnaire for professionals can be found in Multimedia Appendix 1, and the questions in the questionnaire for patients can be found in Multimedia Appendix 2.

### After the Launch

#### Public Relation

Immediately after the official launch, media attention was sought. One of the authors (EK) had interviews on regional and national radio stations, and articles were published in both regional and national newspapers and in magazines from patient organizations, health professionals, and the university. In addition, many websites (eg, Voeding Nu [34], Gezondheid [35], Foodlog [36], Onderzoekers [37]) paid attention to the launch of the website on nutrition and cancer. Each time a question was answered on the website, a notice was sent out via Twitter.

#### Submitted Questions

After the launch, visitors of the website could submit questions. After submitting the question, the person received a standard email explaining the answering procedure: a literature search is performed, the answer is read by a team of experts, and then it is sent to the submitter of the question. The questions were answered by nutritional scientists and dieticians and reviewed by members of the Dutch Dietetic Oncology Group. When necessary, advice was sought from other experts. Questions about specific personal and unique situations received a direct and personal answer. If the patient needed more personal feedback or additional guidance, referral to a dietician or to the treating physician was made. Questions or opinions from people with strong beliefs regarding nutrition and cancer were directly answered by the nutritional scientists and registered dieticians and were not placed on the website. In these answers, an explanation was given that only scientific evidence from sufficient observational studies, meta-analyses, reviews, and RCTs was used to formulate answers, in combination with animal, in vitro, and case studies. General questions on cancer were redirected to other sources of information, such as to the website of the Dutch Cancer Society [11]. General questions about nutrition were redirected to The Netherlands Nutrition Centre [38]. Questions and answers on nutrition and cancer were placed on our website.

#### Categorization of Questions

Visitors of the website who asked a question did not have to sign in or make a profile. Instead, they had to submit a form in which their name, email address, and some questions had to be filled in. To gain insight into the information needs of the visitors of the website, the questions were analyzed. Each form and question, with its matching answer, were read and imported to a Microsoft Access database (Microsoft, Redmond, WA, USA). Questions were categorized and ordered independently by 3 of the authors (MRV, SB, and AMAA). The categories were “products promoting health” and “products harming health/increasing cancer risk.” In addition, 3 periods were defined, namely, “prevention of cancer,” “nutrition during treatment,” and “nutrition after treatment.” Information on the number of question submitters and the number of questions per submitter was recorded and calculated.

#### User Statistics

Google Analytics was used to collect information about the number of page views, the number of visitors, and the length of stay.

## Results

### Prelaunch

#### Overview

Feedback was provided on content and site design; in addition, improvements were also suggested. Based on the results of the pretest, improvements in design, content, and navigation were made before the official launch of the website.

#### Feedback From Patients

Fifty-six patients started the questionnaire, and 38 patients (68%) filled in the complete questionnaire. As can be seen in Table 1, 15 respondents (27%) had visited a website on nutrition and cancer before the launch of our website or a general search on Google. Ten (24%, 10/41) patients did not find the information in our website to be completely clear. Their reactions were “I can’t find which diet is important for my sort of cancer”, and “I do not understand the relationship between apricot kernels and nausea.” Twenty-five patients (45%) thought the website was not complete: “There are only 5 types of cancer”, “What types of food should I avoid?”, “Information is missing”, “it is not completely clear how many nutrients I need to keep my body healthy and fit”, and “You can see the website is not finished yet”. Of all of the patients who completed the questionnaire, 28 (74%) would recommend the website to others. The reasons for not recommending the website were “There is not enough new information on this website”, “I do not see the difference between the website of the Dutch Cancer Society and this website”, and “I hope there will be referrals from other websites, as I do not think you will find this website without a referral”.

**Table 1:** Results of pretest by cancer patients

Question number	Question	Yes	%	No	%	Other	%	Total
Q2	Did you visit other websites on nutrition and cancer?	15	27	41	73			56
Q6	Is it clear in a glimpse what the website is about?	31	76	10	24			41
Q7	Is it clear in a glimpse who the owner of the website is?	17	40	26	60			43
Q9	Does the website meet your expectations?	26	65	14	35			40
Q12	What do you think of the chosen font? Is it clear?	36	92	3	8			39
Q13	What do you think of the colors used? Are they pleasant?	34	87	5	13			39
Q14	What is your opinion of the selection of the pictures? Are they pleasant?	34	87	5	13			39
Q16	Is the layout clear?	34	87	5	13			39
Q17	Can you easily find what you are looking for?	34	87	5	13			39
Q20	Do you think the texts on the website are comprehensible?	37	95	2	5			39
Q23	Do you think the website is complete?	20	53	1	3	17	45	38
Q24	Is it clear where you can ask questions?	29	76	9	24			38
Q27	Does the website look reliable?	33	87	5	13			38
Q28	Would you visit this website?	32	84	6	16			38
Q29	Would you recommend the website to others?	28	74	3	8	7	18	38

For most respondents, the first impression of the website was a positive experience: “clear”, “fresh”, and “looks reliable”. Improvements were suggested as follows: “smaller images”, “not only young and healthy individuals on the images”, “Such a website suggests that you can alter your disease with nutrition, while it actually is about altering your diet to cope with your cancer”, and “Always mention the source, where did you find the answer to the question?”. Suggestions on the structure of the website were also given: one suggestion was “More links”, while another respondent suggested “More scrolling options instead of links”.

#### Feedback From Health Care Professionals and Communication Experts

Fifty professionals from 5 groups (communication specialists, dieticians, nutritional scientists, staff of the World Cancer Research Fund and the Dutch Cancer Society, and patient advocates) were contacted; 23 professionals (46%) provided feedback on the website. They provided comments about its layout, which were similar to the feedback of the patients: “the images are too large”, “the front page is not clear”, and “I do not understand the navigational options of the website”. In addition, comments were made on the question form: “I cannot find your question form”. Other comments included positive remarks on the total look of the website: “I like the vibrant colors and the positive feel of the website”, and “the texts are easily accessible and comprehensible”.

Their general comments on content were as follows: “Why do you only mention the 5 most common types of cancer?”, “Where can I find a full reference list?”, and “I was looking for information for professionals, a pity it is not there”. Opinions differed on the difficulty level of the texts. Some respondents said the level of the texts was appropriate, whereas others commented that the texts were too difficult for the general public.

**Figure 1:**
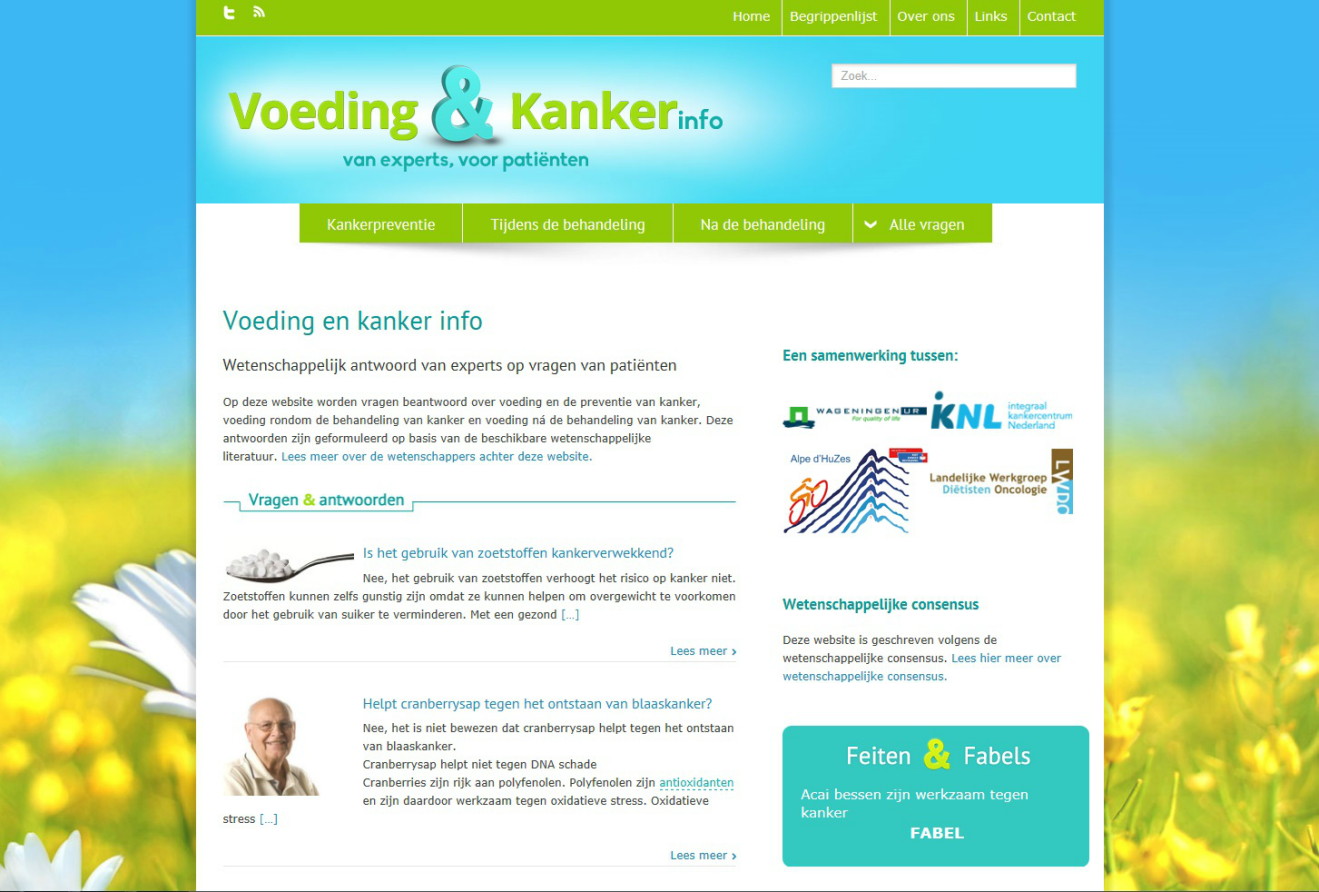
Home page of the website (May 2014).

As a result of all the feedback, a clear distinction was made between the different phases: before treatment (primary prevention), during treatment, and after treatment. Nondiet-related information on tumor types was deleted, because this can be found on other websites [11]. Categorization by the 5 major tumor types was no longer used. Alterations were made to the logo, the font size of the website, the pictures used, and the navigation options. Additional information on nutrition and cancer was uploaded to the website before the launch. The comprehensiveness of the texts was tested [39] and adjusted in such way that all texts matched the B1 level of the Common European Framework [40]. A preview of the home page of the website just before the launch is shown in Figure 1.

### After the Launch

#### Public Relation

**Figure 2:**
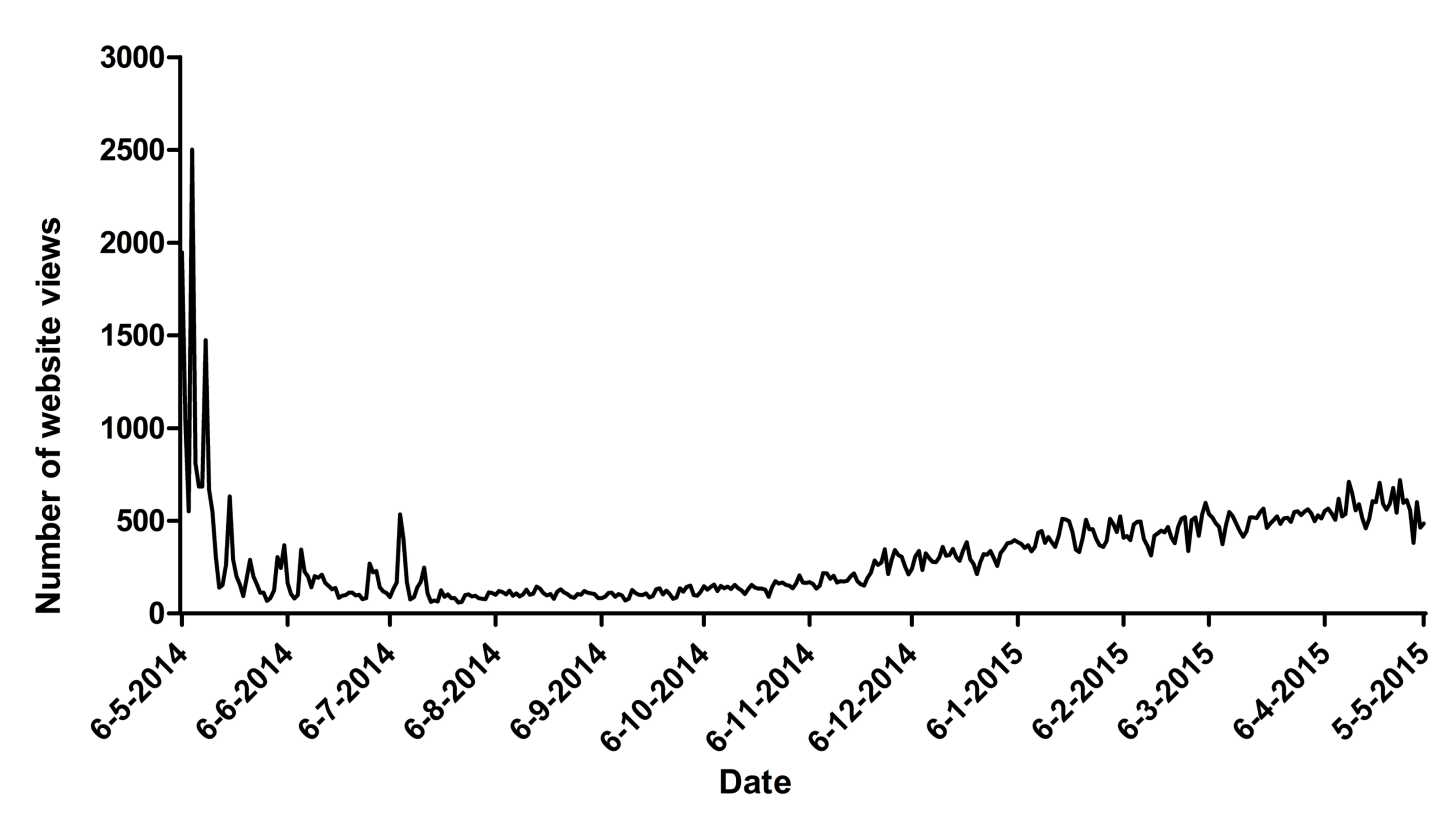
Website views per month.

The results of the public relation activities were reflected in the number of views of the website. Right after the launch in May 2014, the level of media attention was high, and this is reflected in the large number of website views seen in Figure 2. The peaks in May reflect the media attention in the national newspaper Metro and radio interviews, and the peak in July reflects a Facebook post of the National Cancer Institute promoting the website. From December onward, business cards were handed out by dieticians and oncology nurses, and the website was actively promoted at several large conferences, which is reflected by a steady rise in website views and users.

#### User Statistics

**Figure 3:**
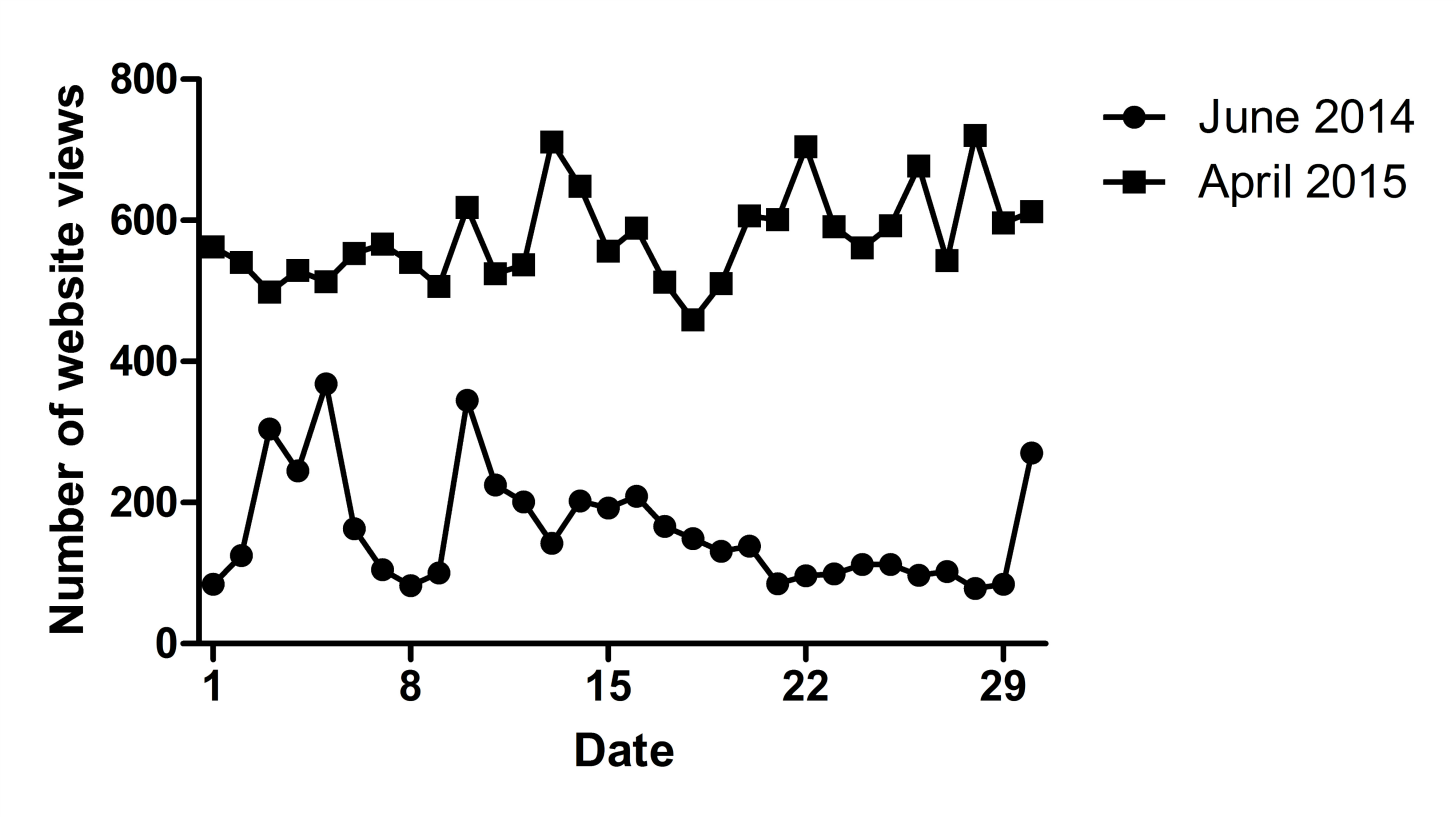
Comparison of website views in June 2014 and April 2015.

Between May 6, 2014, and May 5, 2015, 322,627 page views, 2.9 pages per visit, 90,111 visitors, a length of stay of 2.17 minutes per visit, and 109,596 website views were registered; there was an average of 7509 visitors, 26,885 page views, and 9133 website views per month. An increase can be seen in the number of website views, as shown in Figure 3. As much as 88.33% (n=96,817) of website views were from The Netherlands, 6.74% (n=7391) came from Belgium, and the remaining (4.91%; n=5388) came from non-Dutch speaking countries.

#### Information on Question Submitters

In total, 338 people submitted 404 individual questions. A majority of people sent in 1 question (n=299, 88.4%). Thirty-nine people (12%) who submitted questions asked more than 1 question: 25 submitters asked 2 questions, 9 submitters asked 3 questions, 3 people asked 4 questions, 1 person asked 7 questions, and 1 person submitted 9 questions. Question submitters were not asked for feedback on the website, because we had not informed the visitors of the website that they could be contacted. Therefore, to make sure the privacy of the question submitters was preserved, no user satisfaction survey was performed.

#### Submitted Questions

In the 1st year of the website launch, 404 questions were submitted via the contact form. Detailed information on these questions can be found in Table 2.

**Table 2:** Categorized questions submitted to the website in the first year after the launch.

	Prevention	During treatment	After treatment	Other questions	Total
Products promoting health	64	135	9		208
Products harming health/increasing cancer risk	31	53	5		89
Other topics				107	107
Total	95	188	14	107	404

As can be seen in Table 2, most of the questions were on factors promoting health, both to prevent cancer and during the treatment of cancer.

Representative examples of questions on nutritional factors to prevent cancer, possibly promoting health were “Is biological fruit better?”, “Does the intake of dairy products affect my cancer risk?”, “Can a product such as chia seeds prevent cancer?”, and “What do you think of the use of curcuma to prevent cancer?”. Examples of questions on nutritional factors to prevent cancer, possibly harming health were “Does drinking coffee increase my cancer risk?”, “Is it true that people with a low cholesterol level have an increased cancer risk?”, “Does omega 6 increase cancer risk?”, and “Do artificial sweeteners increase my risk of getting cancer?”.

Questions on nutritional factors during cancer treatment, possibly promoting health were “Can you advise me how to season my foods, to improve taste?”, “Is it known whether broccoli helps to treat my cancer?”, “Can I use probiotics during my cancer treatment?”, and “Are red fruits good to use for breast cancer patients?”.

Questions on nutritional factors during cancer treatment, possibly harming health were “Is it true that sugar feeds my tumor?”, “Is it harmful to eat soy products during my breast cancer treatment?”, and “Is it true that green tea interferes with my antitumor treatment?”.

The questions on nutritional factors after cancer treatment, possibly promoting health were “What can I eat now that my stoma is removed to prevent the recurrence of my cancer?”, “Will eating soy products along with my tamoxifen use help to prevent the recurrence of my breast cancer?”, and “What nutrition can you advise to prevent the recurrence of prostate cancer?”. Questions on nutritional factors after cancer treatment, possibly harming health were “Does cheese harm you when you had breast cancer?”, “Can you drink wine if you are using tamoxifen after breast cancer?”, “Can using curcuma after melanoma treatment harm my health?”, “Does consuming sugar increase my risk of cancer recurrence?”, and “Can I use fruit and yoghurt together after being cured of colorectal cancer?”.

The majority of submitted questions were on specific foods or food components, not on complaints. However, when looking at the questions visited at the website, the top 10 were mostly filled with questions regarding complaints related to their disease, such as nausea, dry mouth, or diarrhea. In addition, 61 questions were requests for business cards.

After 1 year, 121 articles could be found on the website. In these 121 articles, 238 questions could be answered. In total, 372 questions (N=404; 92.0%) were answered. Expert advice was sought on the topics of diabetes/sugar and cancer, vitamin D and cancer, the interaction of foods and nutrients, and pharmaceuticals. The response time to questions was 1 day for the standard response, and the median response time was 3 days (range 0-280 days) for a detailed answer.

## Discussion

### Principal Findings

Before the official launch, the website was designed (based on feedback and suggestions), pretested, and adjusted according to the provided feedback: extra questions and answers were added, the subdivision by tumor type was removed, and alterations were made to the logo, the font size, the pictures used, and the navigation options. In the 1st year after the launch, a total of 404 questions were submitted on nutrition and cancer. Most of the questions were on food products promoting health, both in the prevention of cancer and during the treatment of cancer. A total of 90,111 people visited the website during this period.

When investigating the submitted questions, it was noticed that there are a lot of opinions on nutrition and cancer, which are not completely evidence based. These beliefs often arise from the results of in vitro and animal studies, but are not confirmed in human studies. Therefore, most of the answers to commonly asked questions on the website start with “No, there is no scientific evidence.” This is due to the fact that only studies in humans were used as evidence, because research only conducted in animals and in in vitro studies cannot be directly translated into humans. Besides the evidence-based answers, some answers are based on best practice-based evidence. Questions on complaints are mostly answered according to best practice-based evidence, whereas questions on specific nutrients are provided with evidence-based answers.

The very limited results from in vitro or animal studies sometimes lead to hypes in the media. Some examples are specific foods that people believe could prevent cancer, support cancer treatment, or promote general health (curcuma, chia seeds, etc). A large number of people follow this hype, which is reflected in the number of questions on these food products that were posted on the website. The question arises regarding why a large number of people use these products to prevent cancer or to support cancer treatment, despite the fact that their use is not evidence based. It might be possible that people do not want to put much effort into following healthy lifestyle advice, and therefore, they look for supplements or specific foods that can support their treatment. Indeed, a high number of questions about dietary supplements that might support cancer treatment or might decrease cancer risk were posted on the website.

There was a discrepancy between the topics of the submitted questions and the top 10 questions visited on the website. Therefore, it is likely that the questions on complaints were already answered by the content of the website, and fewer questions had to be asked on this topic.

### Limitations

In the prelaunch test, the nonresponse might have been due to the fact that patients were asked to fill out the questionnaire while receiving their chemotherapy. Furthermore, open questions lead to the highest number of dropouts: “What is your first impression of this website?” and “Is it clear at first sight what information can be found on this website?”

The visitors were not asked for feedback after the launch of the website. However, in a focus group, the patients were asked for their opinions of the website. These patients were glad the website was launched, and they were able to find the information that they were looking for. In their opinion, the website was clear, informative, and complementary to the online information available at that point.

As mentioned earlier, to preserve privacy, no information on the sex and age of visitors was recorded. Therefore, no conclusions can be drawn on the characteristics of the visitors. Because the average time spent on the website was 2.17 minutes, and the visitors visited 2.9 pages on average, we assume that the visitors really searched for information on nutrition and cancer and did not visit the website by accident. However, it is unknown whether these visitors are cancer patients, relatives, health care professionals, or members of the general public. When looking at the type of questions submitted to the website, most of the questions were on the prevention of cancer and nutrition during treatment. This might suggest that both the general public and cancer patients visit the website. The lack of questions on nutrition after treatment could be due to the fact that once people are cured from cancer, they do not want to read about cancer anymore, so these people will not visit the website. Another reason could be that cancer survivors do not think about cancer in relation to tertiary prevention, or that the submitter of the question makes no distinction between primary or tertiary prevention.

The response time in answering questions varied widely for different questions. This was due to the vast number of questions submitted following the launch, and the fact that there were so many questions that needed extensive research. Therefore, the response time was much longer than anticipated. Because it is not appropriate to make people wait for so long, especially in palliative settings, this needed to be resolved. Extra personnel were hired, and the answering methods were optimized: standard answers were formulated that could be adjusted for specific personal situations. The response time has decreased since these measures were taken: most questions are answered within 2 weeks. In addition, questions were submitted that already could be found on the website; these readers were referred to the specific question and answer on the website. This also decreased the response time.

To reach a larger number of potential users of the website, beginning in December 1, 2014, business cards were distributed via oncology nurses, dieticians, and oncologists to inform patients about the existence of the website. This led to an increased number of visitors to the website and eventually to more questions.

### Comparison With Prior Work

Globally, there are more websites on nutrition and cancer; however, our website is the only Dutch website solely on nutrition and cancer where people can ask questions. Internationally, there are other websites, such as that of The Cancer Nutrition Center [41] and the American Society of Clinical Oncology [42]. However, the focus of these websites is exclusively on practical information for the patient, and no scientific evidence is presented. Therefore, our website has a unique concept.

In The Netherlands, there are no other websites on the full scope of cancer and nutrition to prevent cancer, or during treatment and after treatment. Therefore, our website was compared with the nutritional part of The Dutch Cancer Society website [11] and with the Dutch Lung Cancer Information Center website [43].

When comparing the user statistics of our website with the user statistics of the nutritional part of The Dutch Cancer Society [11], we noted some differences. Our website had 7500 visitors per month versus 1250 visitors per month on the nutritional section of the other website. However, the length of stay on our website was substantially shorter: 2.19 minutes on our website, compared with 7.63 minutes on the nutritional part of The Dutch Cancer Society website. The country of origin of the visitors was comparable for both websites. The Dutch Cancer Society website has not seen a change in the number of visitors since the launch of our website [44]. A reason for this might be that our website attracts different people than those visiting the nutritional part of The Dutch Cancer Society website or that people visit both websites. It might also be that people are looking for information on The Dutch Cancer Society website and accidentally visited the part about nutritional information, instead of specifically searching for information on nutrition and cancer. In the past, people might have used other, less reliable websites in addition to their potential use of The Dutch Cancer Society website. Since the launch of our website, they have access to evidence-based information on nutrition and cancer and the opportunity to ask questions about nutrition and cancer.

Some differences were also noted when comparing the results of our website with the results of the website of the Dutch Lung Cancer Information Center [43]. First, the number of questions was substantially higher for the lung cancer website (57 versus 34 questions/month). The relative difference in the number of questions may be due to the topic of both websites. The Lung Cancer website focused on medical questions, which were answered by medical specialists. In comparison, a smaller number of questions could be expected for our website, because it only focuses on nutrition and cancer, thereby targeting only a smaller group of people. Another difference between the 2 websites is that the Lung Cancer website was able to register the identity and sex of its visitors. This information was not recorded on our website. A similarity between both websites is that most visitors only asked 1 question.

### Recommendations for Future Website Developers

Based on the experience from developing this website, the following recommendations are provided to future website developers:

- Start with an assessment of the information needs within the target group. For instance, a patient panel or patient focus groups can be used to explore wishes and demands regarding the future website.
- Before launching the website, test for its readability, usability, completeness, etc. Ask potential users/target group (ie, patients, health care professionals) and communication experts for feedback.
- Make it possible to register user information of the website visitors using cookies, to be able to further tailor the website to your visitors’ demands.
- Do not underestimate the man power needed to maintain a website driven by visitors’ questions. Be sure that there is enough man power to respond quickly to patients’ questions.
- Make use of printed press and social media channels to promote your website to the target group, so your website is used to its full potential.

### Conclusions

The Voeding en Kanker website provides access to evidence-based, best practice-based, and eminence-based information on nutrition and cancer. As can be concluded from the number of questions submitted and the number of visitors to the website, in comparison with the number of visitors to the nutritional part page of The Dutch Cancer Society website and the Dutch Lung Cancer Information Center website, our website fills a gap in the provision of information about nutrition and cancer. Future work includes ongoing improvement of the website by answering questions and responding to current events more quickly. The website will be used to respond to actual news events; additionally, a section with recipes for cancer patients suffering from alterations in their taste and a section especially focused on health care professionals will be developed.

## Appendix

Multimedia Appendix 1 The questions asked in the questionnaire for professionals.

Multimedia Appendix 2 The questions in the questionnaire for patients.

## Abbreviations

RCT: randomized controlled trial
