# Supplementary material for: Development of a Website Providing Evidence-Based Information About Nutrition and Cancer: Fighting Fiction and Supporting Facts Online
Source: JMIR Res Protoc. 2015 Sep 8;4(3):e110. doi: 10.2196/resprot.4757 (PMC4704902; doi:10.2196/resprot.4757)
Supplement: Multimedia Appendix 1 [file resprot_v4i3e110_app1.pdf]

Multimedia appendix 1

Questions we asked healthcare professionals and communication experts

What is your first impression of the website?

What is the look of the website?

How can we improve the appearance of the website?

What is your opinion about the amount of text, the level of the texts, the font, the use of colours and the selection of images?

Do you have any suggestions for improvement?

Is the layout of the website appropriate, is the navigation convenient, and can you find what you are looking for?

What would you want to change about the website, and what can be improved from the point of view of a communication expert?
