# Supplementary material for: Development of a Website Providing Evidence-Based Information About Nutrition and Cancer: Fighting Fiction and Supporting Facts Online
Source: JMIR Res Protoc. 2015 Sep 8;4(3):e110. doi: 10.2196/resprot.4757 (PMC4704902; doi:10.2196/resprot.4757)
Supplement: Multimedia Appendix 2 [file resprot_v4i3e110_app2.pdf]

Multimedia appendix 2

Pre-test questionnaire we sent to the cancer survivors.

Q1 Before you visit www.voedingenkankerinfo.nl, can you elaborate on what information you expect to find there?

Q2 Did you visit other websites on nutrition and cancer before visiting this website?

Q3 What is your first impression of the website?

Q4 What do you think of the look and feel of the website?

Q5 How can we improve the look and feel of the website?

Q6 Is it clear in a glimpse what the website is about?

Q7 Is it clear in a glimpse who is the owner of the website?

Q8 The information displayed on the website is… new/partly new/familiar… to me.

Q9 Does the website meet your expectations?

Q10 If the website does not meet your expectations: what expectations did you have?

Q11 Do you have any suggestions for improvements or adjustments to the website?

Q12 What do you think of the chosen font? Is it clear?

Q13 What do you think of the colours used? Are they pleasant?

Q14 What is your opinion of the selection of the pictures? Are they pleasant?

Q15 Do you have any suggestions with regard to the images?

Q16 Is the layout clear?

Q17 Can you easily find what you are looking for?

Q18 Do you have any suggestions to improve the structure of the website?

Q19 What is your opinion of the amount of text on a page?

Q20 Do you think the texts on the website are comprehensible?

Q21 What do you think of the use of language on the website?

Q22 If the use of language is too difficult or too easy, do you have any suggestions for improvement?

Q23 Do you think the website is complete?

Q24 Is it clear where you can ask questions?

Q25 If you take the website as a whole, what attracts your attention in a positive way?

Q26 If you take the website as a whole, what attracts your attention in a negative way?

Q27 Does the website look reliable?

Q28 Would you visit this website?

Q29 Would you recommend the website to others?

Q30 If you have any final remarks, please post them here.
